# 370. Examining the Relationship Between SARS-CoV-2 PCR Cycle Threshold, Disease Severity and Epidemiologic Trends

**DOI:** 10.1093/ofid/ofab466.571

**Published:** 2021-12-04

**Authors:** Jessica Penney, Amanda Jung, Benjamin Koethe, Shira Doron

**Affiliations:** 1 Tufts Medical Center, Boston, Massachusetts; 2 Tufts University School of Medicine, Somerville, Massachusetts

## Abstract

**Background:**

Real-time reverse transcriptase PCR (rRT-PCR) has become the primary method for detection of SARS-CoV-2. Specific measurements of cycle threshold (Ct) values can give an estimate of viral load. Previous studies have shown temporal trends in Ct values, which could be used to predict the phase of the pandemic. This study’s goal was to examine the relationship between Ct and disease severity, as well as Ct trends.

**Methods:**

Testing was performed using the Abbott M2000 SARS-CoV-2 assay. Data was collected for 262 SARS-CoV-2 positive patients from March-May 2020. Kruskal-Wallis testing was performed to determine differences in median Ct based on age, gender, race and ethnicity. To determine relationship between symptom onset and clinical severity with Ct, linear and logistic regression were performed.

**Results:**

The majority of the patients had mild to moderate disease. Average time since symptom onset was 5.9 days, and 92% were symptomatic. Figure 1 demonstrates the distribution of Ct by disease severity at time of testing. There was no significant difference in cycle threshold by sex, age, race or ethnicity. Figure 2 shows weekly mean cycle threshold by total new cases in Massachusetts to reflect temporal trend of Ct and cases. In the multivariable linear regression model, Ct increased with days since symptom onset (P< 0.001). Cycle threshold was inversely associated with disease severity in multivariable logistic regression though (OR 1.06, 95%CI 1.01-1.11, p=0.03), even when controlling for time since symptom onset.

Figure 1. Distribution of Ct by disease severity at time of SARS-CoV-2 testing

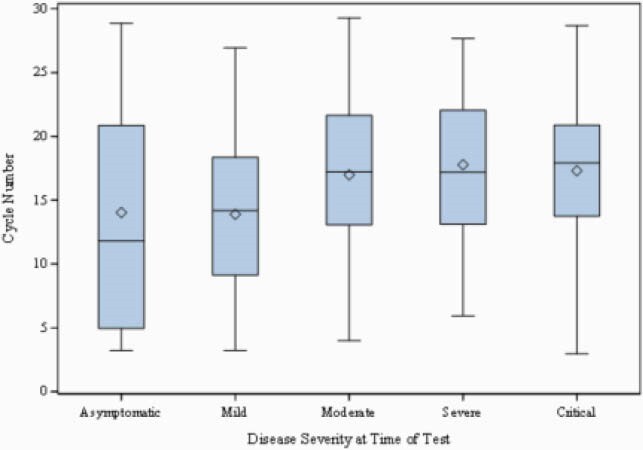

Boxplot demonstrating distribution of Ct by disease severity at time of testing. There was no significant difference between groups.

Figure 2. Weekly Mean Cycle Threshold by Total New MA Cases

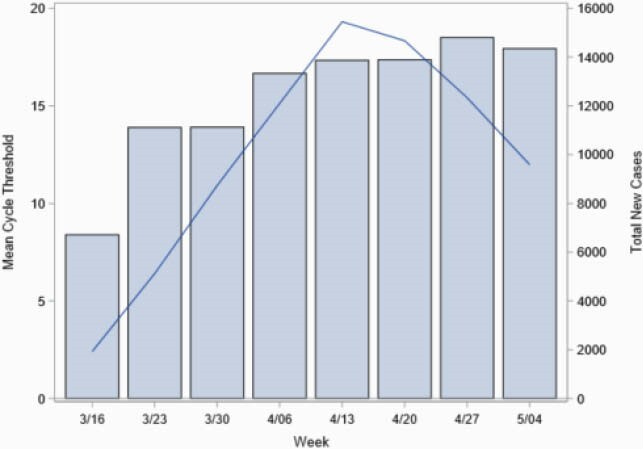

Line represents mean Ct over time period included in this study overlaid on total new cases in Massachusetts. Lower Ct were seen in the course as cases were increasing which peaked as cases stabilized.

**Conclusion:**

Cycle threshold increased with time since symptom onset, consistent with prior data showing increasing Ct from time since infection due to decreasing viral replication. This study showed an inverse relationship between cycle threshold and disease severity, which differs from previous studies which demonstrated higher odds of progression to severe disease and mortality with lower Ct. This finding may reflect the disease severity associated with the secondary inflammatory phase of SARS-CoV-2 seen later in the disease course, although there was only moderate correlation between Ct and time since symptom onset. Further research is needed to better understand the role of Ct in predicting clinical severity of SARS-CoV-2 infections.

**Disclosures:**

**All Authors**: No reported disclosures

